# The mediating roles of workplace support and ethical work environment in associations between leadership and moral distress: a longitudinal study of Canadian health care workers during the COVID-19 pandemic

**DOI:** 10.3389/fpsyg.2023.1235211

**Published:** 2023-09-29

**Authors:** Rachel A. Plouffe, Anthony Nazarov, Ann M. Heesters, Chandlee C. Dickey, Laura Foxcroft, Fardous Hosseiny, Tri Le, P. Andrea Lum, Maede S. Nouri, Patrick Smith, J. Don Richardson

**Affiliations:** ^1^MacDonald Franklin Operational Stress Injury Research Centre, London, ON, Canada; ^2^Schulich School of Medicine and Dentistry, Western University, London, ON, Canada; ^3^Department of Psychology, University of Dundee, Dundee, United Kingdom; ^4^Department of Psychiatry and Behavioural Neurosciences, McMaster University, Hamilton, ON, Canada; ^5^Dalla Lana School of Public Health, University of Toronto, Toronto, ON, Canada; ^6^The Institute for Education Research (TIER), Unviersity Health Network, Toronto, ON, Canada; ^7^Atlas Institute for Veterans and Families, Ottawa, ON, Canada; ^8^Parkwood Institute, London, ON, Canada

**Keywords:** leadership, moral distress, healthcare, workplace ethics, workplace support

## Abstract

**Introduction:**

The COVID-19 pandemic has resulted in heightened moral distress among health care workers (HCWs) worldwide. Past research has shown that effective leadership may mitigate potential for the development of moral distress. However, no research to date has considered the mechanisms by which leadership might have an influence on moral distress. We sought to evaluate longitudinally whether Canadian HCWs’ perceptions of workplace support and ethical work environment would mediate associations between leadership and moral distress.

**Methods:**

A total of 239 French- and English-speaking Canadian HCWs employed during the COVID-19 pandemic were recruited to participate in a longitudinal online survey. Participants completed measures of organizational and supervisory leadership at baseline and follow-up assessments of workplace support, perceptions of an ethical work environment, and moral distress.

**Results:**

Associations between both organizational and supervisory leadership and moral distress were fully mediated by workplace supports and perceptions of an ethical work environment.

**Discussion:**

To ensure HCW well-being and quality of care, it is important to ensure that HCWs are provided with adequate workplace supports, including manageable work hours, social support, and recognition for efforts, as well as an ethical workplace environment.

## Introduction

Throughout the COVID-19 pandemic, health care workers (HCWs) have consistently faced occupational stressors that place them at risk for experiencing psychological distress. For example, HCWs have exhibited acute or chronic symptoms of depression, anxiety, post-traumatic stress, and burnout resulting from the pressure to maintain high quality care while working within a constrained healthcare system (Heath et al., 2020; Sirois and Owens, 2021). HCWs are also frequently exposed to situations in which they are obliged to work in ways that are incongruent with their professional ethical values, such as caring for a greater number of patients than is recommended, witnessing compromised patient care, as well as working outside the scope of their duties on reassignment (Rosa et al., 2020; Norman et al., 2021). The psychological symptoms associated with these morally fraught situations, including frustration, guilt, anger, anxiety, and physical sensations, are characteristics of moral distress (Hamric et al., 2012). Moral distress is formally defined by instances in which the HCW knows what is ethically correct, yet encounters institutional limitations that limit the pursuit of the morally appropriate action (Jameton, 1984).

### Leadership and psychological well-being in health care workers

Despite the occupational difficulties associated with working in healthcare during the COVID-19 pandemic, some protective factors against psychological and moral distress have been identified (Ahmed et al., 2020, 2021; Rosa et al., 2020; Zhao et al., 2020; Plouffe et al., 2021). For example, inclusive leadership, defined by leaders who express appreciation for employees’ contributions and include them in decision-making practices, has been positively related to well-being, as well as reductions in psychological manifestations of distress in nurses during the COVID-19 pandemic (Ahmed et al., 2020, 2021; Zhao et al., 2020). Similarly, in a sample of Canadian nurses, those who perceived their leaders as encouraging and appreciative of their contributions reported higher levels of psychological well-being at work (Nelson et al., 2014). The empirical associations between positive leadership characteristics and psychological outcomes cited in recent research are unsurprising, as inclusive and authentic leaders encourage open communication, clearly outline rules and regulations, support others’ learning and development, and adopt an inclusive approach to workplace activities (Malila et al., 2018; Kuknor and Bhattacharya, 2022). On the other hand, patient-facing HCWs who perceived a lack of support from leadership were more likely to report that they experienced moral distress during the COVID-19 pandemic (Ness et al., 2021). Similarly, moral distress was exacerbated among a sample of emergency HCWs in the United States during the COVID-19 pandemic when leaders were unprepared, distant, or unsupportive of employees (Blanchard et al., 2022). In their qualitative assessments, these HCWs reported that leaders were often reactive to challenges as opposed to proactive and did not focus on providing staff with appropriate resources or effective communication (Blanchard et al., 2022). These findings further emphasize the significance of positive leadership characteristics throughout the COVID-19 pandemic, as HCWs provided essential services to the public during this public health emergency, and ensuring their safety and well-being contributes to lower burnout and job turnover (Rosa et al., 2020; Tabur et al., 2022).

The importance of effective leadership is in line with the Job Demands-Control Model of organizational psychology (Karasek Jr, 1979; Matthews and Ritter, 2017), such that the interplay of high job-related demands and lack of perceived control (i.e., degree of employee autonomy in making workplace decisions) exerts influence on negative well-being outcomes. Based on this model, the most stressful situations will be elicited when HCWs perceive their work environment as both demanding and out of their control. Thus, effective leader behavior, including strong communication and preparation for potential moral stressors, may serve to mitigate potential for moral distress and other psychological outcomes.

### Leadership and health care worker psychological distress: mediating variables

Although positive leadership characteristics frequently contribute to lower levels of psychological distress and higher levels of well-being in HCWs, these associations are often influenced by external variables that are related both to leadership and psychological outcomes. For example, multiple studies have shown that links between effective leadership and psychological distress were mediated by psychological safety (Zhao et al., 2020; Ahmed et al., 2021), workplace motivation (Fernet et al., 2015), workplace spirituality (McKee et al., 2011), sense of community (McKee et al., 2011), and “work life” areas (workload, perceptions of control, reward, sense of community, fairness, and value congruence; Laschinger et al., 2015). It is thus evident that authentic and inclusive leaders serve to promote a positive workplace environment and support their staff in overcoming workplace challenges, which in turn reduces levels of psychological distress among HCWs.

### Ethical workplace environment and workplace support as potential mediators of associations between leadership and health care worker moral distress

Previous empirical studies have found negative associations between effective leadership and HCW psychological distress, as well as mediators of these relations (Zhao et al., 2020). In addition, it is evident that supportive leadership in a HCW setting is related to reductions in moral distress (de Veer et al., 2013; Ness et al., 2021; Plouffe et al., 2021). For example, Ness et al. (2021) demonstrated that among patient-facing nurses in the United States, support from leadership, limited accessibility of personal protective equipment, and frequently changing workplace protocols contributed to higher levels of moral distress. Despite the growing empirical literature reflecting predictors of HCW moral distress, mediating factors underlying the links between leadership and moral distress have not been identified in prior research. There is a growing need to assess potential mediators in associations between leadership and moral distress during the COVID-19 pandemic, as understanding elements influencing the extent of moral distress experienced by HCWs is essential for protecting both their own welfare and that of their patients by maintaining high quality patient care. If potential mediators are identified, then workplace protocols can also be established that serve to enhance protective factors associated with moral distress. Lastly, no studies to date have longitudinally evaluated links between leadership and moral distress. These investigations are imperative to ensure that potential causal mechanisms of moral distress are accurately identified and are not confounded by the use of cross-sectional data. Two potential mediators of these longitudinal associations in HCWs include perceptions of workplace support and an ethical work environment.

Consistent with the strong ethical issues associated with moral distress in HCWs (Jameton, 1984), reduced perceptions of an ethical workplace environment are related to higher levels of moral distress (Corley et al., 2005; Whitehead et al., 2015; Rathert et al., 2016; Lamiani et al., 2017; Plouffe et al., 2021; Riedel et al., 2022). Similarly, effective leadership in health care is related to perceptions of an ethical workplace climate (Özden et al., 2019). This is unsurprising, as authentic, inclusive leaders work to promote transparent workplace communication, a safe environment, rapport with patients and employees, and employee development (Avolio et al., 2004; Alilyyani et al., 2018), all of which are relevant to fostering an ethical workplace environment, defined as a workplace in which ethical values guide behavior and are reflected in the organization’s strategies, structures, and processes (McDaniel, 1997; Corley et al., 2005). Given these connections, it is plausible that HCWs’ perceptions of an ethical work environment will mediate associations between organizational management/supervisory leadership and moral distress. Similarly, based on prior research investigating mediators in the associations between leadership and HCW psychological distress (McKee et al., 2011; Laschinger et al., 2015), as well as relations between workplace support and moral distress (Plouffe et al., 2021), it is plausible that higher levels of workplace support enacted by organizational and supervisory leadership contribute to reductions in HCW moral distress. Specifically, authentic leadership styles are related to components of workplace support including HCW feelings of competency at work, support in decision-making, appreciation of efforts, social connection, fairness of work allocation, and alignment of individual and workplace values (Laschinger et al., 2015), which in turn may reduce levels of moral distress in HCWs.

### Objectives and hypotheses

Heightened moral distress in HCWs inevitably contributes to poor mental and physical health outcomes, as well as employment-related outcomes, including low job satisfaction, dissatisfaction with quality of patient care, and burnout (de Veer et al., 2013; Lamiani et al., 2017). Under the Job Demands-Control Theory (Karasek Jr, 1979; Matthews and Ritter, 2017), when HCWs experience high workplace demands and low control over tasks and workload (as is the case in a morally-distressing environment), they are more likely to encounter negative well-being outcomes. To provide adequate supports and prevent these deleterious outcomes for both HCWs and their patients, it is crucial for researchers and HCW organizations to work toward a comprehensive understanding of protective factors associated with moral distress.

To fulfill the need to assess causal relations between relevant workplace variables and moral distress, our aim was to longitudinally assess perceptions of workplace support and ethical work environment as mediators in the relations between both organizational and supervisory leadership and moral distress in Canadian HCWs. Based on findings from prior studies (Özden et al., 2019; Ness et al., 2021; Plouffe et al., 2021), we hypothesize that workplace support, ethical work environment, and leadership will be significantly and positively correlated, whereas moral distress will be negatively correlated with these workplace variables. Based on previous mediation findings related to psychological distress (Laschinger et al., 2015), we further hypothesize that perceptions of workplace support and an ethical work environment will significantly mediate associations between organizational/supervisory leadership and moral distress.

Notably, although some studies have evaluated relations between leadership and moral distress (Ness et al., 2021; Plouffe et al., 2021), these studies have been cross-sectional in nature. In order to accurately draw causal conclusions regarding workplace support and ethical work environment as mediators underlying the relations between leadership and moral distress, it is important to assess variables longitudinally at distinct time points. Thus, we assessed variables of interest across three separate time points during the COVID-19 pandemic.

## Methods

### Participants and procedure

A total of 237 English and 2 French-speaking HCWs across Canada were included as participants in this longitudinal study. HCWs were defined as individuals “who provide health treatment and advice based on formal training and experience, or who work to directly support those providers in a clinical setting (Liu et al., 2021).” To be eligible to participate, HCWs were required to be at least 18 years of age or older and employed (or previously employed) as a HCW in Canada between the beginning of the COVID-19 pandemic and the baseline data collection end date (December 31, 2020).

This study was approved by the Health Science Research Ethics Board at Western University (#115894) and Lawson Health Research Institute (#9968). A detailed protocol of study procedures is available (Liu et al., 2021). We used a convenience snowball sampling approach to recruit Canadian HCWs. Participants were recruited *via* word of mouth, emails to professional networks, an online advertisement through Lawson Health Research Institute, social media advertisements, and a participant recruitment website: ParticipAid.co. Participants who provided informed consent at baseline completed a battery of measures online *via* the survey-hosting platform, Research Electronic Data Capture. Participants were able to select English or French versions of the study. Baseline data collection began on June 26, 2020, and ended on December 31, 2020. Participants were then invited *via* email to complete a series of follow-up surveys every 3 months following completion of baseline surveys for a total of 18 months.

Although a total of 1,926 Canadian HCWs completed the baseline surveys, only those who completed the specific measures of interest across at least three consecutive time points, reported that they retained the same workplace position over the three time points (i.e., did not change or leave their job), and did not switch from full-time to part-time hours (or vice versa) were included in this study. Thus, the final *n* for this study was 239. *A priori* sample size for our parallel mediation models was calculated using the application Monte Carlo Power Analysis for Indirect Effects (Schoemann et al., 2017) run *via* R Version 1.3.1073 (R Development Core Team, 2020) with 5,000 replications. Using previous effect sizes reported by Plouffe et al. (2021), to achieve a power value of at least 0.80, 220 participants were required to detect a significant indirect effect with workplace support as a mediator, and 57 participants were required to detect a significant indirect effect with ethical work environment as a mediator. Thus, our sample size of 239 was adequate for our mediation models.

### Measures

#### Ethical work environment

We used the Ethics Environment Questionnaire (EEQ; McDaniel, 1997) to measure health care workers’ perceptions of the ethical environments in their organizations at the second time point (3 months following first time point). Items on the 20-item EEQ are evaluated on a 5-point scale (1 *= strongly disagree* to 5 = *strongly agree*). Higher mean scores on the EEQ are indicative of higher ethical workplace environment perceptions, with scores ranging from 1 to 5. Past research has supported the reliability of the EEQ for use in HCW samples (e.g., Cronbach’s α = 0.93; McDaniel, 1997; Corley et al., 2005). In her initial investigation, McDaniel (1997) found that the EEQ demonstrated strong construct, content, and criterion validity in a sample of acute care HCWs.

#### Moral distress

Moral distress was assessed at the third time point (6 months following first time point) using the 27-item Measure of Moral Distress for Healthcare Professionals (MMD-HP; Epstein et al., 2019). The MMD-HP items are measured on 4-point scales that assess the frequency (0 = *never,* 4 = *very frequently*) and distress severity (0 = *none,* 4 = *very distressing*) associated with experiences known to contribute to moral distress in a HCW setting. Consistent with recommendations by Epstein et al. (2019), aggregate scores were calculated by multiplying frequency (0–4) by distress (0–4) item scores and summing the multiplied item scores (range = 0–16). Scores on the MMD-HP range from 0 to 432, with higher scores representing more severe moral distress. Past research has supported the reliability of the MMD-HP (e.g., Cronbach’s α = 0.93; Epstein et al., 2019). The MMD-HP has also shown strong dimensional and convergent validity in nurses and physicians (Epstein et al., 2019).

#### Workplace support and leadership

We assessed HCWs’ perceptions of their workplaces’ supervisory and organizational management leadership, as well as workplace support using subscales of the Pandemic Experiences and Perceptions Survey (PEPS; Leiter, 2020). The subscales measure workplace support (i.e., “work life” subscale) using seven items on a scale ranging from 1 = *strongly disagree* to 5 = *strongly agree*, and supervisory and organizational management leadership separately (5 items each) on scales ranging from 1 = *not at all* to 5 = *frequently, if not always*. For this study, leadership was assessed at the first time point and workplace support was assessed at the second time point (3 months after the first time point). Workplace support items reflect, for example, sustainability of workload, perceptions of competence, social support, and fairness of workplace decisions. Leadership items reflect, for example, whether supervisors and organizational management expressed hope for success, identified actions to improve workplace capabilities, and helped HCWs to be safe. Higher mean scores across the three subscales indicated stronger perceptions of workplace support and leadership, with scores ranging from 1 to 5. The reliability of the PEPS has been supported in recent research (e.g., Cronbach’s α = 0.86; Smith, 2022), and its dimensionality and convergent validity have been supported in past research in HCW samples (Plouffe et al., 2021; Smith, 2022).

### Data analytic strategy

Bivariate correlations and descriptive statistics, including means, standard deviations, Cronbach’s alphas, McDonald’s omegas, and skewness and kurtosis values were calculated using IBM Statistical Package for Social Sciences Version 27 (IBM Corp, 2019). Given the dimensional nature of study variables, constructs were assessed as individual differences on a continuum and score cut-offs were not applied.

In accordance with best practices in structural equation modeling (Kline, 2015), we conducted measurement models using MPlus Version 8 (Muthén and Muthén, 1998–2017) to assess model fit and the relations between leadership, ethical work environment, workplace support, and moral distress. Two separate measurement models were conducted for both organizational and immediate supervisory leadership using the maximum likelihood estimator. We conducted two separate models because past research implementing the PEPS organizational and supervisory leadership demonstrated poor model fit due to overlap in item wording (Plouffe et al., 2021). Specifically, modification indices indicated that items with identical wording across the two types of leadership demonstrated strong residual correlations (e.g., How did you experience (*organizational/supervisory*) leadership during the pandemic period… Identify specific actions that would improve our capabilities?). Therefore, we chose not to include the two variables in the same model due to the strong overlap between the constructs resulting from common method variance, which may result in biased estimates and non-replicability.

Organizational leadership, supervisory leadership, ethical work environment, and workplace support were entered as latent variables with items as indicator variables (except for ethical work environment). Consistent with recommendations outlined by Matsunaga (2008), we used parcels as indicators for the 20-item EEQ to stabilize parameter estimates for measures with a larger number of items. In addition, because the MMD-HP is a formative measure, we used one indicator variable to represent moral distress (Bollen and Bauldry, 2011).

Next, we evaluated the hypothesis that workplace support and ethical work environment would significantly mediate the associations between organizational/supervisory leadership and moral distress. Two structural equation models were tested with organizational management leadership and supervisory leadership as outcome variables. We also present results for additional structural equation models with mediators entered separately in Supplementary Table S1. Mediation models were conducted using 1,000 bias-correcting bootstrap confidence intervals (CIs). If the 95% bias-corrected bootstrapped CIs do not contain zero for the indirect effect, this reflects the presence of mediation (Preacher and Hayes, 2008).

Model fit for the measurement and structural models were evaluated according to recommendations outlined by Browne and Cudeck (1992) and Hu and Bentler (1999). Specifically, root mean square error of approximation (RMSEA) indices close to 0.06 reflect good model fit, between 0.07 and 0.08 reflect acceptable fit, between 0.08 and 0.10 reflect marginal fit, and greater than 0.10 reflect poor fit (Browne and Cudeck, 1992; Hu and Bentler, 1999). Comparative fit indices (CFIs) and Tucker-Lewis indices (TLIs) between 0.90 and 0.95 reflect acceptable fit, and 0.95 or larger reflect strong fit (Browne and Cudeck, 1992; Hu and Bentler, 1999). Lastly, we considered the relative chi-square (chi-square values divided by the degrees of freedom) less than 3 to be adequate (Carmines and McIver, 1981).

We used full information maximum likelihood to estimate missing data. There were no missing values for moral distress. The maximum number of missing data points across the leadership scales was one item per case (*n*_cases_ = 3 for organizational; *n*_cases_ = 6 for supervisory). Seven participants were missing scores on ethical work environment, and one participant was missing scores on the workplace support scale. However, where individuals were missing scores on one mediator, they had no missing values on the other mediator.

## Results

### Participant sociodemographic and workplace information

Participants most commonly reported working as nurses (*n =* 101, 42.3%), physicians (*n* = 14, 5.9%), physical therapists (*n* = 12, 5.0%), social workers (*n* = 8, 3.3%), and personal support workers (*n* = 8, 3.3%). Other professions included, for example, paramedics, radiographers, respiratory therapists, health administrators, psychologists, and pharmacists, among others. Most participants also reported working full time (*n* = 187, 78.2%), whereas the remaining participants worked part-time positions (*n* = 52, 21.8%). At baseline data collection, most participants also reported that they had been involved in the diagnosis, treatment, or providing care to patients with COVID-19 in the past month (*n* = 137, 57.3%). Additional sociodemographic and workplace information is provided in Table 1.

**Table 1:** Sociodemographic and workplace information.

Variable	Frequency	Percentage (%)
**Age (years)**		
≤25	4	1.7
26–40	76	31.8
41–60	139	58.2
>60	15	6.3
Prefer not to answer	1	0.4
Missing	4	1.7
**Gender**		
Women	211	88.3
Men	20	8.4
Other	1	0.4
Prefer not to answer	4	1.7
Missing	3	1.3
**Marital status**		
Single	47	19.7
Married/common law	153	64.0
Separated	6	2.5
Divorced	24	10.0
Widowed	1	0.4
Prefer not to answer	5	2.1
Missing	3	1.3
**Province/territory**		
Northwest Territories	3	1.3
Newfoundland	2	0.8
New Brunswick	5	2.1
Nova Scotia	5	2.1
Quebec	3	1.3
Saskatchewan	7	2.9
Manitoba	12	5.0
British Columbia	22	9.2
Alberta	23	9.6
Ontario	97	40.6
Missing	60	25.1
**Highest level of education**		
Secondary or lower	1	0.4
Post-secondary or higher	235	98.4
Missing	3	1.3
**Primary job function**		
Administration	21	8.8
Outreach	3	1.3
Research	3	1.3
Direct client/patient care	202	84.5
Other	9	3.8
Missing	1	0.4
**Number of years as a health care worker**		
<6 months	1	0.4
6 months to 1 year	2	0.8
1–5 years	23	9.6
6–10 years	49	20.5
11+ years	163	68.2
Missing	1	0.4
**Percentage direct patient care**		
No direct patient care	12	5.0
1–24%	16	6.7
25–50%	16	6.7
51–74%	33	13.8
75–100%	162	67.8
Missing	–	–
**Workplace setting**		
Private practice	24	10.0
Hospital/community health centre	169	70.7
Other	46	19.2
Missing	–	–

### Descriptive statistics and bivariate correlations

Descriptive statistics, including means, standard deviations, skewness values, kurtosis values, Cronbach’s alphas, and McDonald’s omegas, as well as bivariate correlations, are reported in Table 2. Cronbach’s alphas and McDonald’s omegas were high for all measures, with alphas and omegas ranging from 0.86 (workplace support) to 0.97 (supervisory leadership). Skewness and kurtosis values fell within the recommended range of ±3.00 (Kline, 2015). The mean level of moral distress was reported as 103.29 out of a possible 432. This is consistent with past mean levels of moral distress reported in studies of HCWs (Epstein et al., 2019).

**Table 2:** Descriptive statistics and bivariate correlations.

Measures	α	ω	Skewness	Kurtosis	*M (SD)*	1	2	3	4
1. T1 Organizational leadership	0.92	0.92	−0.04	−0.74	3.09 (1.05)				
2. T1 Supervisory leadership	0.97	0.97	−0.12	−1.16	3.09 (1.26)	0.76			
3. T2 Ethical work environment	0.95	0.95	−0.03	−0.09	3.06 (0.77)	0.60	0.56		
4. T2 Workplace support	0.86	0.86	−0.30	−0.06	3.28 (0.87)	0.53	0.47	0.65	
5. T3 Moral distress	0.95	0.95	0.97	0.68	103.29 (84.03)	−0.44	−0.35	−0.61	−0.52

α = Cronbach’s alpha. ω = McDonald’s omega. T1, Time 1; T2, Time 2; T3, Time 3. All correlations significant at *p* < 0.001.

Organizational and supervisory leadership (measured at Time 1), workplace support, and perceptions of an ethical work environment (both measured at Time 2) were positively and significantly correlated with effect sizes ranging from medium-to-large (Cohen, 1988). Moral distress (measured at Time 3) was negatively and significantly correlated with organizational and supervisory leadership, workplace support, and perceptions of an ethical work environment with medium effect sizes (Cohen, 1988).

### Measurement models

We conducted two measurement models to assess the associations between organizational/supervisory leadership, ethical work environment, workplace support, and moral distress. Fit for the first model with organizational leadership as a predictor was adequate, *χ*^2^(114) = 306.62, *p* < 0.001, χ^2^/*df* = 2.69, CFI = 0.94, RMSEA = 0.08 (90% CI = 0.07, 0.09), *p* < 0.001. Factor loadings were all significant at *p* < 0.001, ranging from 0.34 (workplace support) to 0.93 (ethical work environment). Variables of interest were all significantly correlated at *p* < 0.001, ranging from −0.46 (organizational leadership and moral distress) to 0.76 (ethical work environment and workplace support).

The second measurement model, with supervisory leadership as a predictor, fit the data well, χ^2^(114) = 232.65, χ^2^/*df* = 2.04, *p* < 0.001, CFI = 0.97, RMSEA = 0.07 (90% CI = 0.05, 0.08), *p* = 0.017. Factor loadings were significant at *p* < 0.001, ranging from 0.34 (workplace support) to 0.93 (supervisory leadership). Variables of interest were significantly correlated at *p* < 0.001, ranging from −0.35 (supervisory leadership and moral distress) to 0.76 (ethical work environment and workplace support). Overall, these results support the structure of the hypothesized models of interest.

### Structural model: workplace support and ethical work environment as mediators in association between organizational leadership and moral distress

**Figure 1:**
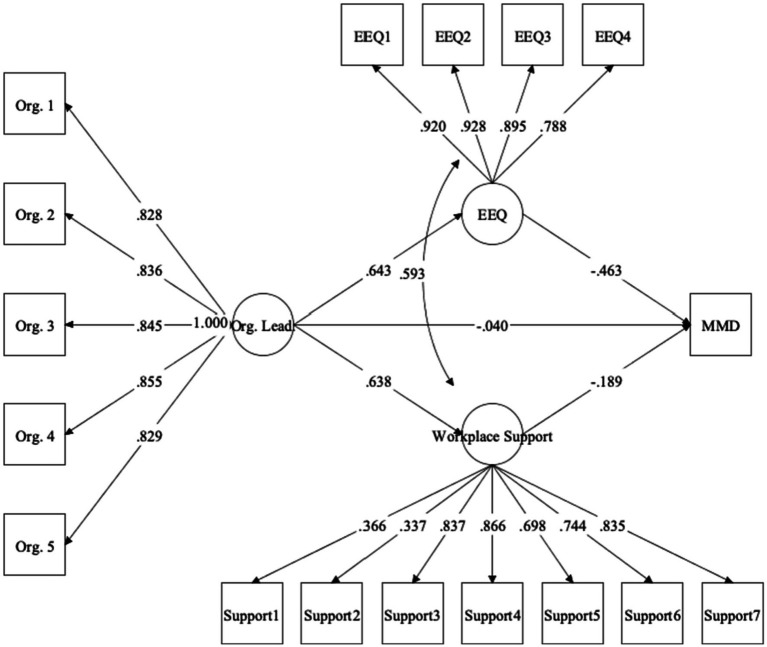
Structural equation model with ethical work environment and workplace support as mediators underlying association between organizational leadership and moral distress. Values represent standardized coefficients. All coefficients significant at *p* < 0.001 except for organizational leadership ➔ moral distress (*p* = 0.623) and workplace support ➔ moral distress (*p* = 0.047). Org. Lead., organizational leadership; EEQ, ethical work environment; MMD, moral distress.

Parameters for the first structural equation model, with workplace support and perceptions of ethical work environment as mediators in the association between organizational leadership and moral distress, are depicted in Figure 1. Indices for this model indicated adequate fit to the data, *χ*^2^(114) = 306.62, *p* < 0.001, *χ*^2^/*df* = 2.69, CFI = 0.94, RMSEA = 0.08 (90% CI = 0.07, 0.09), *p* < 0.001. The total effect was significant (*β* = −0.46, 95% CI = −0.57, −0.31, standard error [SE] = 0.06, *p* < 0.001), indicating that organizational leadership at Time 1 significantly predicted levels of moral distress at Time 3 when other variables were not included in the model. When the Time 2 mediators were introduced, the direct effect became non-significant (*β* = −0.04, 95% CI = −0.21, 0.12, SE = 0.08, *p* = 0.623), supporting the overall presence of full mediation. Consistent with this, the total indirect effect was significant (*β* = −0.42, 95% CI = −0.55, −0.31, SE = 0.06, *p* < 0.001), as well as the specific indirect effect for ethical work environment (*β* = −0.30, 95% CI = −0.44, −0.19, SE = 0.06, *p* < 0.001), for which the 95% CIs did not contain zero. The mediating effect of ethical work environment accounted for 65.22% of the total effect. However, the specific indirect effect for workplace support was only marginally significant (*β* = −0.12, 95% CI = −0.24, −0.01, SE = 0.06, *p* = 0.047), accounting for 26.09% of the total effect. This was likely due to overlapping variance between latent ethical work environment and workplace support (*r* = 0.59, *p* < 0.001), as when only workplace support was included as a mediator, the indirect effect was significant at the 0.001 level (see Supplementary material).

### Structural model: workplace support and ethical work environment as mediators in association between supervisory leadership and moral distress

**Figure 2:**
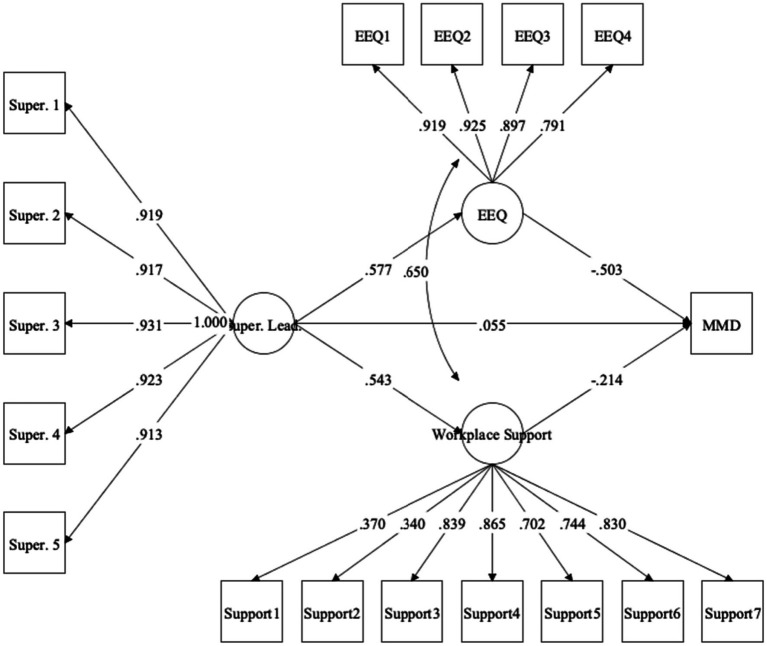
Structural equation model with ethical work environment and workplace support as mediators underlying association between supervisory leadership and moral distress. Values represent standardized coefficients. All coefficients significant at *p* < 0.001 except for supervisory leadership ➔ moral distress (*p* = 0.408). Super. Lead., supervisory leadership; EEQ, ethical work environment; MMD, moral distress.

Parameters for the second structural equation model, with workplace support and perceptions of ethical work environment as mediators in the association between supervisory leadership and moral distress, are depicted in Figure 2. Indices for this model indicated strong fit to the data, *χ*^2^(114) = 232.65, *χ*^2^/*df* = 2.04, *p* < 0.001, CFI = 0.97, RMSEA = 0.07 (90% CI = 0.05, 0.08), *p* = 0.017. The total effect was significant (*β* = −0.35, 95% CI = −0.46, −0.23, SE = 0.06, *p* < 0.001), indicating that supervisory leadership at Time 1 significantly predicted levels of moral distress at Time 3 when other variables were not included in the model. When the Time 2 mediators were introduced, the direct effect became non-significant (*β* = 0.06, 95% CI = −0.07, 0.19, SE = 0.07, *p* = 0.408), supporting the overall presence of full mediation. The total indirect effect was also significant (*β* = −0.41, 95% CI = −0.52, −0.31, SE = 0.05, *p* < 0.001), as well as the specific indirect effects for ethical work environment (*β* = −0.29, 95% CI = −0.42, −0.19, SE = 0.06, *p* < 0.001) and workplace support (*β* = −0.12, 95% CI = −0.22, −0.03, SE = 0.05, *p* = 0.016). The indirect effects for ethical work environment and workplace support accounted for 82.86 and 34.29% of the total effect, respectively. See Supplementary material for supervisory leadership models with separate mediators.

## Discussion

Healthcare workers frequently encounter significant challenges associated with their duties, including working under high-stress conditions, responding to continuously evolving policies and protocols, and managing high workloads, which may elicit exposure to morally distressing situations (Riedel et al., 2022). Identifying the factors that impact levels of moral distress is crucial for safeguarding the well-being of HCWs and upholding quality of patient care. Therefore, the purpose of this research was to evaluate longitudinally whether perceptions of workplace support and ethical work environment mediated associations between organizational and supervisory leadership and moral distress in Canadian HCWs.

In line with our hypotheses, organizational and supervisory leadership, workplace support, and perceptions of an ethical work environment were significantly and positively correlated. This is consistent with past findings indicating that in the context of health care, workplaces with leaders who promote healthy, transparent, and stimulating environments also tend to promote a sense of community, fairness, control over one’s duties, and workplace ethics (Wong and Cummings, 2009; Cummings et al., 2010; Van der Heijden et al., 2017; Alilyyani et al., 2018). On the other hand, moral distress was significantly and negatively related to perceptions of organizational and supervisory leadership, workplace support, and perceptions of an ethical work environment. This finding was also unsurprising, as HCWs who encounter situations in which their moral standards and values are compromised have reported a negative ethical climate and lack of support from leadership, frequently changing guidelines and regulations, and reduced workplace quality of life in past research (Corley et al., 2005; Ness et al., 2021).

As hypothesized, we found that an ethical work environment and high degree of workplace support significantly mediated the associations between organizational management/supervisory leadership and moral distress. Although the indirect effect for workplace support was only marginally significant for organizational leadership, this was due to overlapping variance with ethical work environment, as it was significant when included as a mediator on its own. Our findings illustrated that both leadership at the organizational management and supervisory level are important predictors of subsequent HCW perceptions of an ethical climate and support in the workplace, and that these factors ultimately contribute to reductions in moral distress. Given these significant mediation findings, it is evident that HCWs’ mental health and levels of moral distress are affected not only by their personal interactions with organizational management and supervisors, but also by the ability of leaders to create a healthy and supportive environment (Nelson et al., 2014). This aligns with research suggesting that leadership, characterized by, for example, identifying actions necessary to improve workplace protocols, confidence in the team, promoting safe practices, and open, timely communication (Leiter, 2020) generally contributes to better quality of care for patients (Sfantou et al., 2017), HCW psychological well-being (Nelson et al., 2014), and HCW job performance (Montano et al., 2017). By promoting ethical practices in the workplace, supporting organizational decisions that align with HCWs’ values, and appreciating HCWs’ efforts, leaders in health care are promoting positive workplaces environments that contribute to their employees’ positive mental health (Nelson et al., 2014; Alilyyani et al., 2018). It is evident that the success of health care organizations begins with its leaders (Rosa et al., 2020). These are influential actors who generate the conditions required to maintain high quality patient care and reduce HCW moral distress.

### Practical implications

As the first longitudinal study to assess perceptions of an ethical work environment and workplace support as mechanisms underlying the relations between leadership and moral distress in Canadian HCWs, this research has important implications. With the knowledge that leaders can mitigate or exacerbate moral distress through workplace support and ethical factors, specific steps can be taken to improve the HCWs’ quality of work-life. For example, leaders should continue to support HCWs by clearly and transparently communicating potential moral challenges faced in their roles, modeling positive workplace behaviors and work-life balance during the onboarding stage, and providing staff members with mental health and workplace-related supports (Phoenix Australia – Centre for Posttraumatic Mental Health and the Canadian Centre of Excellence – PTSD, 2020; Rosa et al., 2020). By openly communicating about potential exposure to moral stressors and justifications for solutions to morally challenging situations, leaders should actively listen to and acknowledge HCWs’ experiences while reinforcing their sense of purpose and seeking solutions to the problems they faced. For example, an effective solution to reducing moral stressors related to staffing shortages would involve rotating HCWs between high- and low-stress duties to allow them to recover from potential moral distress or burnout and to spread workloads more equitably (Phoenix Australia – Centre for Posttraumatic Mental Health and the Canadian Centre of Excellence – PTSD, 2020).

Based on our findings and recommendations reported by Rosa et al. (2020), it is also increasingly important to offer support from ethics consultants and to provide open channels to discuss potential ethical issues within the workplace, as well as potential mitigation strategies. This will serve to alleviate potential negative mental health outcomes associated with morally distressing events. Given their role in maintaining psychological well-being and reducing moral distress, top leadership should also be made accessible to HCWs to discuss workplace concerns (Rosa et al., 2020). For example, leaders should regularly meet with HCWs to discuss best practices, barriers to quality patient care, HCW concerns, and problem-solving techniques (Rosa et al., 2020).

Lastly, at the managerial level, health care organizations should implement clear evidence-based protocols to evaluate what should be done when faced with moral or ethical dilemmas (Phoenix Australia – Centre for Posttraumatic Mental Health and the Canadian Centre of Excellence – PTSD, 2020), such as ensuring access to the expertise of those with experience in the realm of ethical deliberation. This ensures that HCWs are not burdened with the task of determining how to navigate difficult morally distressing situations that may arise, such as scarcity of health care resources or staff. These practices are especially important during periods of disruption, as was the case with the COVID-19 pandemic, as HCWs have more frequently encountered situations that do not align with their values such as working outside of their general competencies, witnessing poor quality of care due to shortages of resources or staff, and not respecting patient autonomy (Plouffe et al., 2021). If HCW leaders at the managerial and supervisory levels adopt and promote ethical behaviors and provide support to their staff, their employees are likely to experience lower levels of moral distress and enjoy a positive work environment that fosters growth, development, and patient-centred care.

### Limitations and future directions

Despite the strengths of this study, including the use of longitudinal data across a diverse range of health care occupations, some limitations must be considered. First, although we collected longitudinal data from a considerable number of HCWs across Canadian provinces and territories, our sample may not accurately reflect the broader population of Canadian HCWs. Specifically, our sample consisted predominantly of women and nurses. Future studies should employ stratified sampling methods to ensure adequate representation of the Canadian HCW population.

An additional limitation reflects the convenience sampling method of data collection. We recruited participants using word of mouth, emails to professional networks, social media advertisements, and a participant recruitment website (Liu et al., 2021). It is, therefore, plausible that HCWs were more likely to sign up to participate if they had encountered stressors in the workplace and wanted an outlet for sharing their perspectives. Consequently, the sample could have disproportionately included individuals who were experiencing greater distress during the COVID-19 pandemic, as opposed to those who were coping well.

Lastly, our study relied on collecting self-report responses, which may be susceptible to biases such as socially desirable responding. In other words, participants may attempt, either intentionally or not, to present themselves in a favorable light by understating potential mental health symptomatology or workplace issues. Future research should replicate the current study while evaluating responses from both HCWs and their leaders to determine whether the results are invariant across the groups.

### Concluding remarks

This study was the first longitudinal investigation designed to evaluate how perceptions of ethical work environment and support in the workplace mediate the links between leadership and moral distress among Canadian HCWs during the COVID-19 pandemic. In order to prevent long-term moral distress and deliver high quality patient care, is crucial for leaders of HCW organizations to prioritize the well-being of their employees. Effective, transparent, and inclusive communication should be maintained to ensure adherence to core ethical principles, and supports should be implemented to ensure that HCW mental health is sustained.

## Data availability statement

The raw data supporting the conclusions of this article will be made available by the authors, without undue reservation.

## Ethics statement

This study involving humans was approved by Health Science Research Ethics Board at Western University (#115894) and Lawson Health Research Institute (#9968). The studies were conducted in accordance with the local legislation and institutional requirements. The participants provided their written informed consent to participate in this study.

## Author contributions

RP: conceptualization, analysis, and writing – original draft. AN: conceptualization, funding acquisition, writing – editing and reviewing draft, and supervision. AH: conceptualization, resources, writing – editing and reviewing drafts. CD: conceptualization, resources, writing – editing and reviewing drafts. LF: conceptualization, resources, writing – editing and reviewing drafts. FH: conceptualization, funding acquisition, writing – editing and reviewing drafts. TL: project administration, writing – editing and reviewing drafts. PL: conceptualization, resources, writing – editing and reviewing drafts. MN: analysis, writing – editing and reviewing drafts. PS: conceptualization, funding acquisition, writing – editing and reviewing drafts. JR: conceptualization, writing – editing and reviewing drafts, supervision. All authors contributed to the article and approved the submitted version.

## Funding

This project was funded through support of the MacDonald Franklin OSI Research Centre by the St. Joseph’s Health Care Foundation (London, Ontario, Canada) and through a partnership with Atlas Institute for Veterans and Families (Ottawa, Ontario, Canada).

## Conflict of interest

The authors declare that the research was conducted in the absence of any commercial or financial relationships that could be construed as a potential conflict of interest.

## Publisher’s note

All claims expressed in this article are solely those of the authors and do not necessarily represent those of their affiliated organizations, or those of the publisher, the editors and the reviewers. Any product that may be evaluated in this article, or claim that may be made by its manufacturer, is not guaranteed or endorsed by the publisher.
