# Supplementary material for: The mediating roles of workplace support and ethical work environment in associations between leadership and moral distress: a longitudinal study of Canadian health care workers during the COVID-19 pandemic
Source: Front Psychol. 2023 Sep 29;14:1235211. doi: 10.3389/fpsyg.2023.1235211 (PMC10570733; doi:10.3389/fpsyg.2023.1235211)
Supplement: Supplementary file 1 [file Data_Sheet_1.docx]

**Figure S1**

*Structural Equation Model with Ethical Work Environment as Mediator Underlying Association Between Organizational Leadership and Moral Distress*


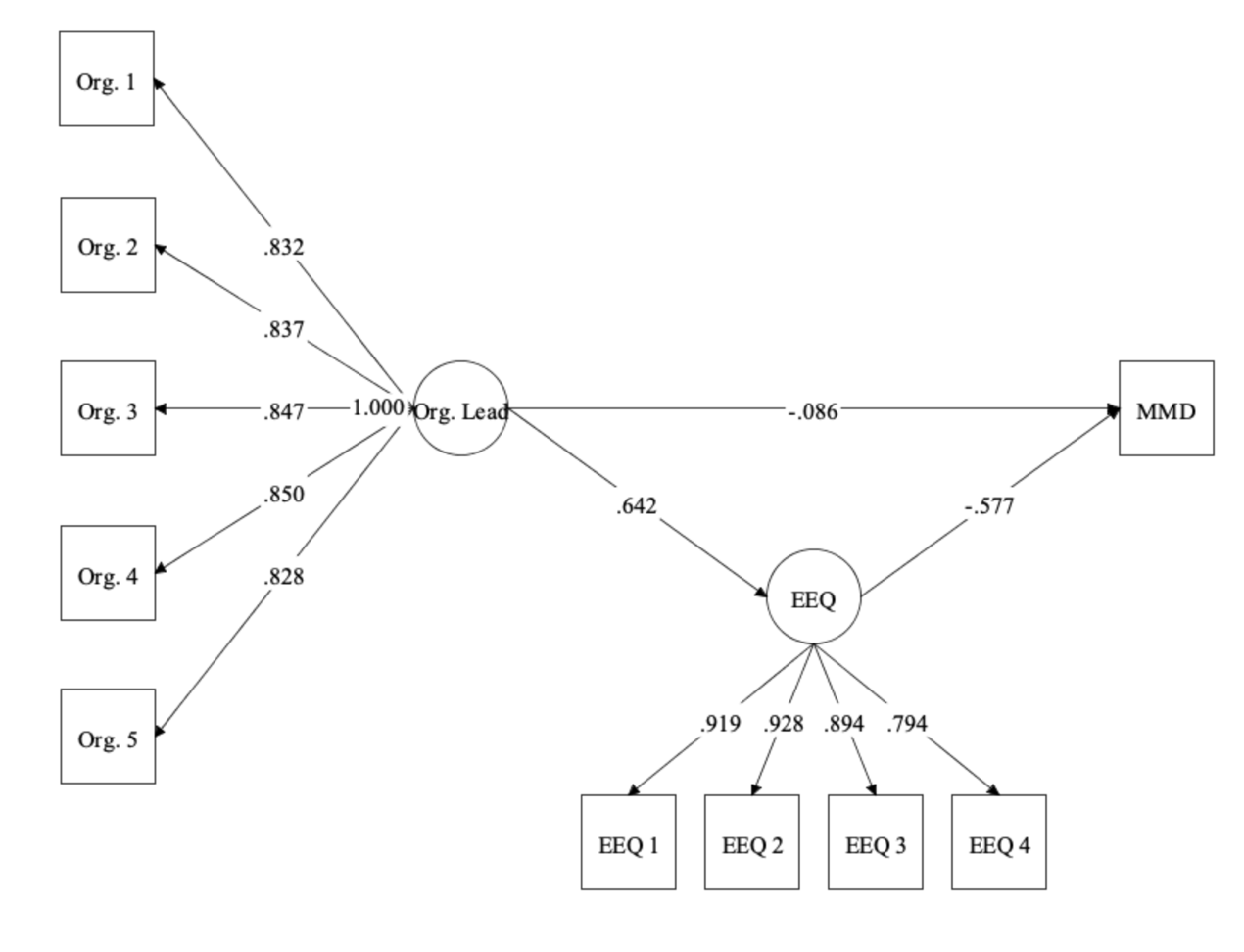


*Note.* Values represent standardized coefficients. All coefficients significant at *p* < .001 except for organizational leadership 🡪 moral distress (*p* = .268). Org. Lead. = organizational leadership; EEQ = ethical work environment; MMD = moral distress. Model fit: χ^2^(33) = 140.57, *p* < .001, CFI = .945, RMSEA = .12 (90% CI = .10, .14), *p* < .001.

**Figure S2**

*Structural Equation Model with Workplace Support as Mediator Underlying Association Between Organizational Leadership and Moral Distress*


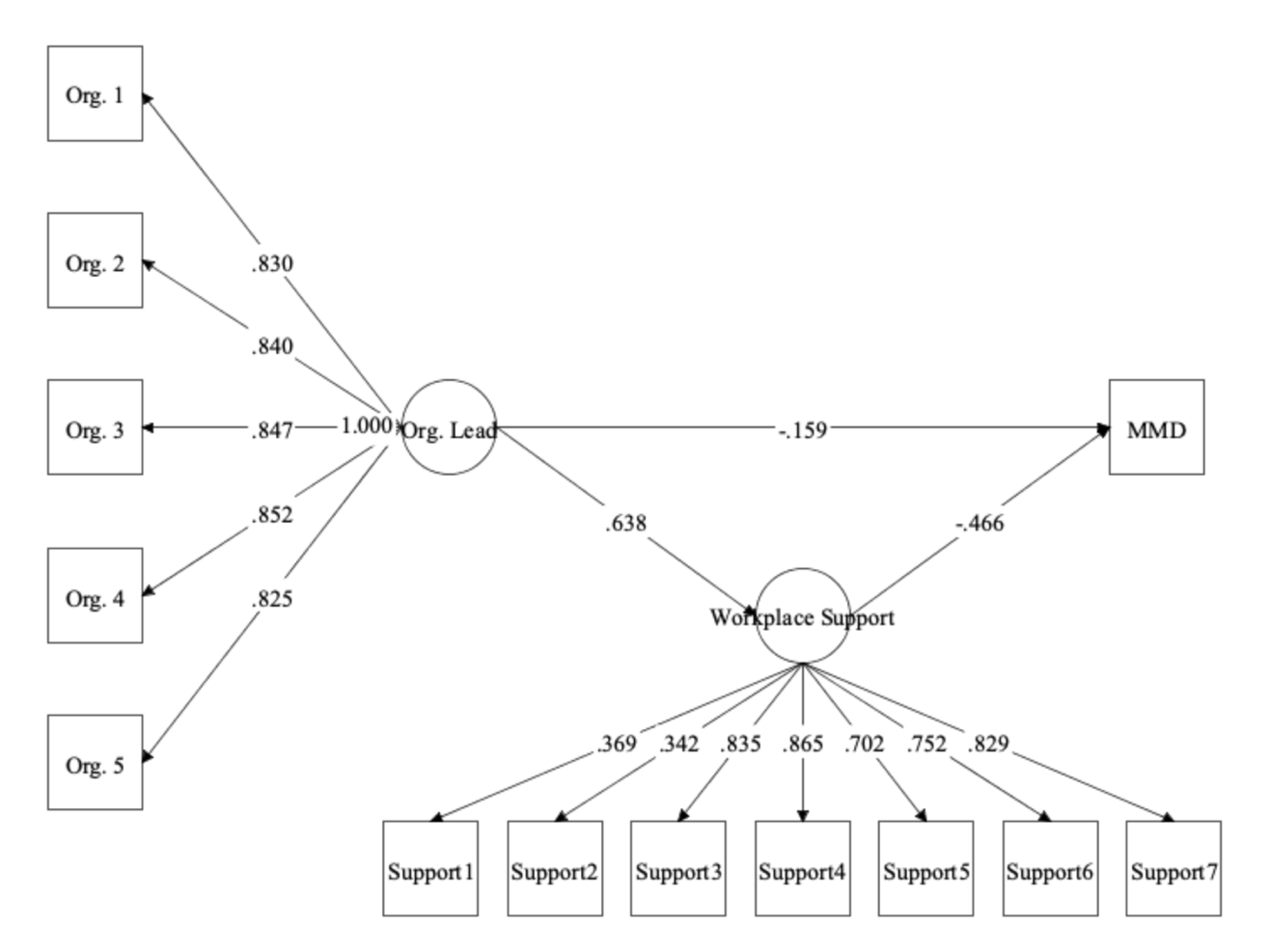


*Note.* Values represent standardized coefficients. All coefficients significant at *p* < .001 except for organizational leadership 🡪 moral distress (*p* = .053). Org. Lead. = organizational leadership; MMD = moral distress. Model fit: χ^2^(63) = 228.52, *p* < .001, CFI = .914, RMSEA = .11 (90% CI = .09, .12), *p* < .001.

**Figure S3**

*Structural Equation Model with Ethical Work Environment as Mediator Underlying Association Between Supervisory Leadership and Moral Distress*


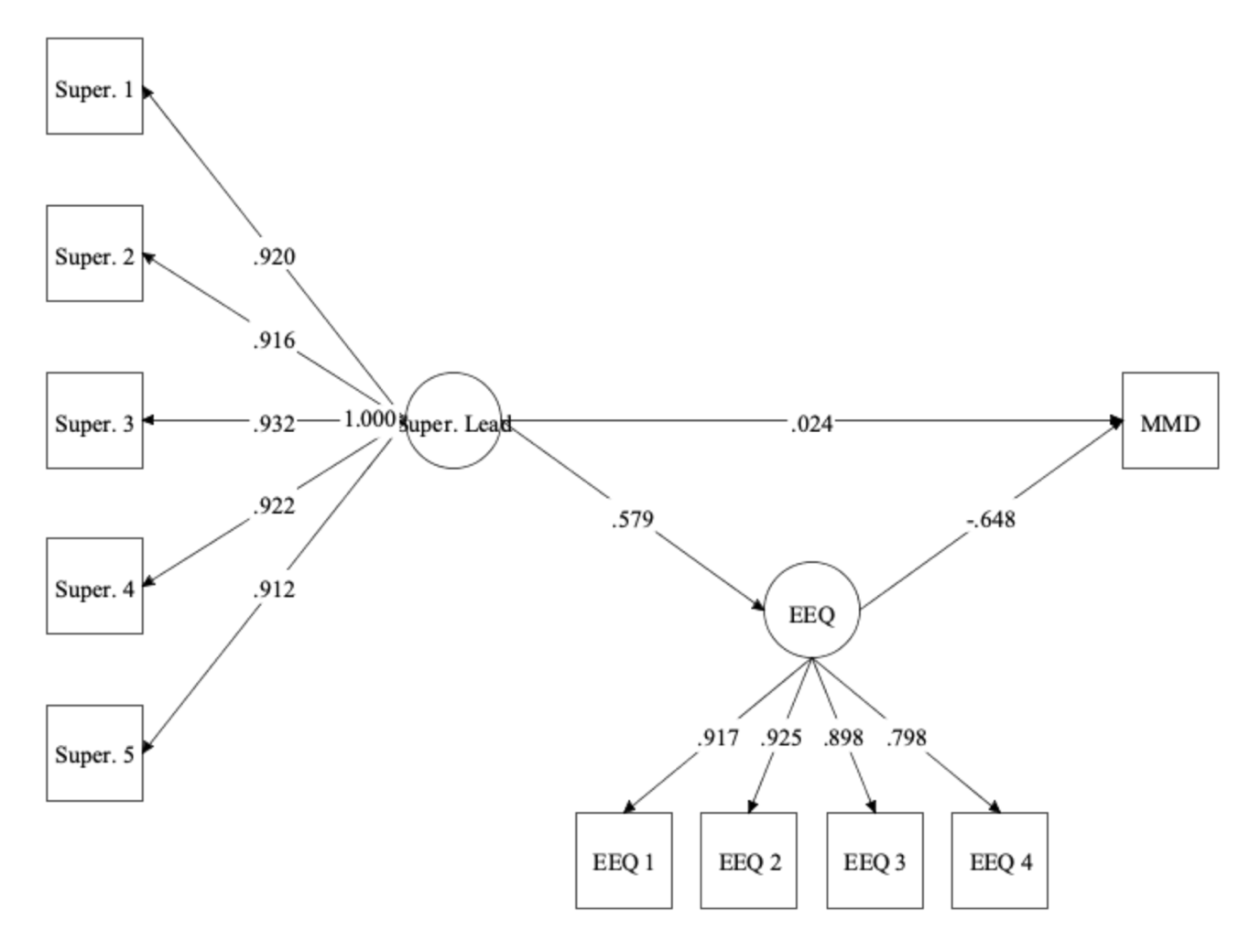


*Note.* Values represent standardized coefficients. All coefficients significant at *p* < .001 except for supervisory leadership 🡪 moral distress (*p* = .716). Super. Lead. = supervisory leadership; EEQ = ethical work environment; MMD = moral distress. Model fit: χ^2^(33) = 78.11, *p* < .001, CFI = .982, RMSEA = .08 (90% CI = .05, .10), *p* = .027.

**Figure S4**

*Structural Equation Model with Workplace Support as Mediator Underlying Association Between Supervisory Leadership and Moral Distress*


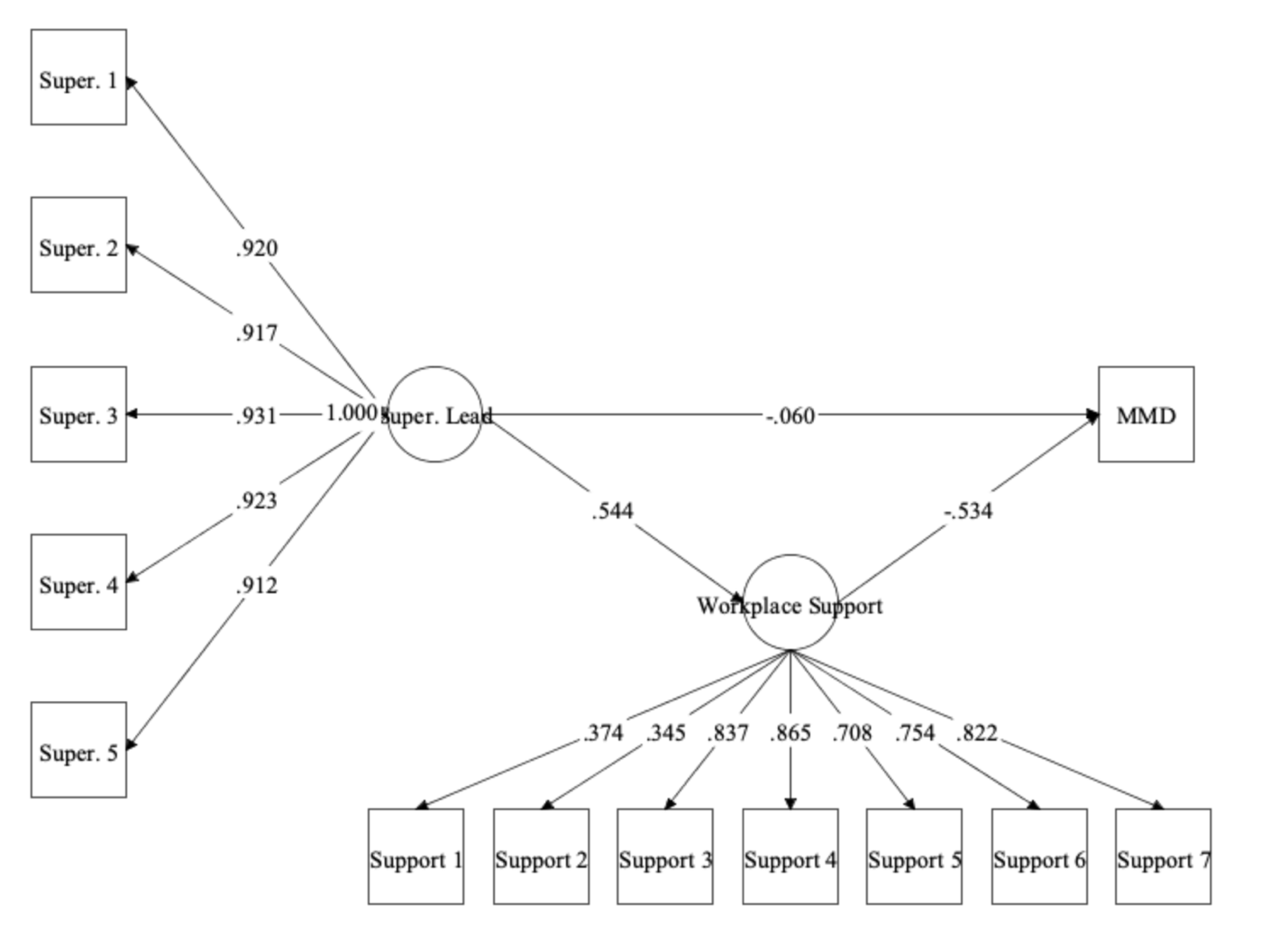


*Note.* Values represent standardized coefficients. All coefficients significant at *p* < .001 except for supervisory leadership 🡪 moral distress (*p* = .361). Super. Lead. = supervisory leadership; EEQ = ethical work environment; MMD = moral distress. Model fit: χ^2^(63) = 143.20, *p* < .001, CFI = .967, RMSEA = .07 (90% CI = .06, .09), *p* = .010.
